# Specific antibody-receptor interactions trigger InlAB-independent uptake of listeria monocytogenes into tumor cell lines

**DOI:** 10.1186/1471-2180-11-163

**Published:** 2011-07-11

**Authors:** Martin Heisig, Alexa Frentzen, Birgit Bergmann, Katharina Galmbacher, Ivaylo Gentschev, Christian Hotz, Christoph Schoen, Jochen Stritzker, Joachim Fensterle, Ulf R Rapp, Werner Goebel

**Affiliations:** 1 Institut für Medizinische Strahlenkunde und Zellforschung (MSZ), Universität Würzburg, Versbacher Straße 8, Würzburg, 97078, Deutschland; 2 Institut für Mikrobiologie, Universität Würzburg, Biozentrum Am Hubland, Würzburg, 97074, Deutschland; 3 Yale University, School of Medicine, Department of Internal Medicine, Section of Infectious Diseases, 300 Cedar Street, New Haven, CT 06520, USA; 4 Genelux Corporation, 3030 Bunker Hill St., Ste310, San Diego, CA 92109, USA; 5 Interlab GmbH, Bayerstr. 53, München, 80335, Deutschland; 6 Abteilung für Klinische Pharmakologie, Klinikum der Universität München, Ziemssenstraße 1, München, 80336, Deutschland; 7 Institut für Hygiene und Mikrobiologie, Universität Würzburg, Josef-Schneider-Str. 2, Würzburg, 97070, Deutschland; 8 Æterna Zentaris, Weismüllerstrasse 50, Frankfurt/Main, 60314, Deutschland; 9 Max Planck Institut für Biochemie, Abteilung für molekulare Biologie, Am Klopferspitz 18, Martinsried, 82152, Deutschland; 10 Max-von-Pettenkofer Institut für Hygiene und Medizinische Mikrobiologie, LMU München, Pettenkoferstr. 9a, München, 80336, Deutschland

## Abstract

**Background:**

Specific cell targeting is an important, yet unsolved problem in bacteria-based therapeutic applications, like tumor or gene therapy. Here, we describe the construction of a novel, internalin A and B (InlAB)-deficient *Listeria monocytogenes *strain (Lm-spa^+^), which expresses protein A of *Staphylococcus aureus *(SPA) and anchors SPA in the correct orientation on the bacterial cell surface.

**Results:**

This listerial strain efficiently binds antibodies allowing specific interaction of the bacterium with the target recognized by the antibody. Binding of Trastuzumab (Herceptin^®^) or Cetuximab (Erbitux^®^) to Lm-spa^+^, two clinically approved monoclonal antibodies directed against HER2/neu and EGFR/HER1, respectively, triggers InlAB-independent internalization into non-phagocytic cancer cell lines overexpressing the respective receptors. Internalization, subsequent escape into the host cell cytosol and intracellular replication of these bacteria are as efficient as of the corresponding InlAB-positive, SPA-negative parental strain. This specific antibody/receptor-mediated internalization of Lm-spa^+ ^is shown in the murine 4T1 tumor cell line, the isogenic 4T1-HER2 cell line as well as the human cancer cell lines SK-BR-3 and SK-OV-3. Importantly, this targeting approach is applicable in a xenograft mouse tumor model after crosslinking the antibody to SPA on the listerial cell surface.

**Conclusions:**

Binding of receptor-specific antibodies to SPA-expressing *L. monocytogenes *may represent a promising approach to target *L. monocytogenes *to host cells expressing specific receptors triggering internalization.

## Background

Bacteria-mediated tumor therapy has been investigated for over a century [1]. The ability of bacteria to colonize malignant tissue has been exploited in different therapeutic approaches [2,3].

The delivery of therapeutic agents by bacteria to the tumor represents a promising approach to eradicate the tumor from the inside [4,5]. A major prerequisite is the specific bacterial colonization of tumor tissue without simultaneous colonization of healthy tissue.

Obligate anaerobic bacteria like *Clostridia *or *Bifidobacteria *colonize solely the anoxic parts of tumors due to their inability to tolerate oxygen [6,7]. For facultative anaerobic bacteria like *Salmonella, Escherichia, Vibrio *or *Listeria*, specific tumor colonization has been described and different therapeutic approaches were investigated [4,8-11]. In general, virulence-attenuated Gram-positive bacterial pathogens, such as *Listeria monocytogenes*, may be better suited for the systemic application of bacteria in tumor therapy as these bacteria lack the LPS of gram-negative bacteria. LPS may induce strong immune reactions culminating in septic shock after release into the blood stream.

*Listeria monocytogenes *(*Lm*) has been successfully studied as carrier for the delivery of DNA and RNA into mammalian cells [12,13]. In this case pathogenicity of the listerial carrier strain was attenuated by the deletion of *aroA *[14]. In contrast to most other applied virulence-attenuated *Lm *strains [10,15,16], the *aroA *mutant possesses all virulence factors, thus enabling the carrier bacteria to invade mammalian cells, escape from the phagosome, and replicate in the cytosol of infected host cells. The intracellular replication rate of the *aroA *mutant was, however, lower compared to the according wild-type strain and the capability of cell-to-cell spread was drastically reduced [14].

The cytosolic life cycle of *Lm *poses an advantage for the delivery of nucleic acids harboring eukaryotic expression cassettes compared to other intracellular bacteria like Salmonella, which reside and replicate in phagosomal compartments. The utilization of *Lm *as a carrier for the direct delivery of prodrug-converting-enzymes and for the introduction of DNA encoding these enzymes into tumor cells *in vitro *was successfully assessed recently [17]. Internalization of *Lm *into non-phagocytic mammalian cells is mainly triggered by the two internalins A and B encoded by the *inlAB *operon [reviewed in 18]. The deletion of *inlAB *thus strongly reduces the ability of *Lm *to actively invade such host cells, but does not change their passive uptake by phagocytic cells.

The targeting of carrier microorganisms to cell surface proteins of specific cells was first performed in viral gene therapy [19]. By genetic fusion of *Staphylococcus aureus *protein A (SPA) to viral coat proteins monoclonal antibodies recognizing specific receptors on the target cells were fixed to the viral surface. Due to the thereby achieved specific virus/cell interaction, uptake of the viral carrier by the selected target cells could be obtained. Alternatively, single chain antibody fragments (scFv) were expressed on the viral surface which - by the interaction with specific receptors on the host cell surface - led to preferential viral infection of the specific target cells as well.

Many tumor cells overexpress specific marker proteins on their surface which include oncoproteins. HER1 (ErbB1) and HER2 (ErbB2), members of the EGFR/HER family, represent such prominent surface proteins [20,21]. Enhanced expression or mutational activation of these cell surface proteins leads to tumor progression and generally correlates with poor prognosis in tumor therapy [reviewed in 22]. Both tumor markers, HER1 and HER2, are specifically recognized by the chimeric/humanized monoclonal antibodies, Erbitux (Cetuximab) and Herceptin (Trastuzumab) which are approved for therapy of colorectal carcinoma and breast cancer, respectively.

Antibody-mediated targeting of bacteria to tumor cells was described so far only for *Salmonella enterica *serovar Thyphimurium expressing a scFv against carcino-embryonic-antigen CEA. Antibody expression resulted in a 2-fold increase of these bacteria in the tumor tissue [23].

As a novel approach we describe in this study the construction of a virulence-attenuated *Lm *strain with deletions in *inlAB *and *aroA *which expresses functional SPA anchored to the cell wall. This strain, when coated with Herceptin or Erbitux, triggered a highly efficient, InlAB-independent internalization into tumor cell lines over-expressing HER1 and HER2, respectively, but not into cell lines lacking these receptors. In a xenograft murine tumor model we could also observe a significant increase in tumor colonization of this *Lm *strain after intravenous injection when the respective antibody was covalently crosslinked to the surface-exposed SPA.

## Results

### Expression of recombinant SPA by internalin A and B deficient *L. monocytogenes *and its correct orientation on the listerial cell surface

A S.au *reus *protein A (SPA)-expressing *Lm *strain was constructed by replacing the non-essential phage integrase/recombinase gene *int *in the genome of the listerial mutant Δ*trpS,aroA,inlA/B *× pFlo-*trpS *by the *spa *gene (encoding the protein A). SPA is controlled by the listeriolysin (*hly*) promoter (P*hly*). The P*hly *carrying DNA fragment contained the signal sequence of *hly *which was fused *in frame *to the *spa *gene. The *spa *gene sequence encodes all five Fc binding domains and the LPXTG motif for sortase-dependent anchoring of the SPA protein to peptidoglycan [24]. The expressed SPA protein thus contains all regions necessary for efficient translocation across the bacterial cell membrane and for anchoring SPA to the cell wall of *Lm*. This *Lm *strain (Δ*trpS, aroA,inlA/B,int*::P*hly*-*spa *× pFlo-*trpS*) is named Lm-spa^+ ^in the following.

**Figure 1:**
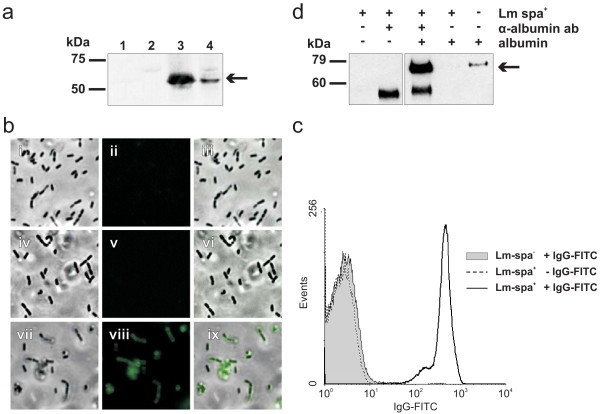
**Expression of *S. aureus *protein A (SPA) on the cell surface of *L. monocytogenes *strain Δ*trpS,aroA,inlA/B,int*::P*hly*-*spa *× pFlo-*trpS *(Lm-spa^+^)**. (**a**) Western blot analysis with polyclonal goat-anti-Protein A antibody of protein extracts from Δ*trpS,aroA,inlA/B *× pFlo-*trpS *(Lm-spa^-^, lanes 1 and 2) and Lm-spa^+ ^(lanes 3 and 4); lanes 1 and 3: cell surface protein extracts; lanes 2 and 4: internal protein extracts. The arrow indicates the position of SPA in the SDS-PAGE. (**b**) Immunofluorescence micrographs showing specific binding of antibody Fc-part to SPA on the surface of Lm-spa^+^. Lm-spa^+ ^were incubated with polyclonal anti-OVA antibody and stained with OVA-FITC protein (vii-ix). Lm-spa^- ^stained with antibody and OVA-FITC (i-iii) and Lm-spa^+ ^stained without antibody but with OVA-FITC protein (iv-vi) were used as negative controls. Phase contrast pictures are shown in the left column; FITC-stained images in middle column; picture overlays in the right column. (**c**) Flow cytometry quantifying the specific Fc-mediated antibody binding to SPA on the surface of *L. monocytogenes *strains. Mid-logarithmic grown bacteria were stained with polyclonal FITC-conjugated rabbit-anti-goat immunoglobulin G (H+L). Grey area indicates strain Lm-spa^-^, while the white area indicates strain Lm-spa^+^. (**d**) Western blot analysis was used for indirect quantitation of protein A on the surface of Lm-spa^+^. 5 × 10^8 ^bacteria were incubated simultaneously with antibody directed against native albumin and an excess of albumin. After incubation bacteria were washed and the amount of albumin bound to the bacteria via antibody was quantified by Western blot analysis with a primary antibody directed against denatured albumin. In the right lane 10 ng of pure serum albumin was applied as control.

Expression of SPA by the constructed *Lm *strains was analyzed by Western blotting using polyclonal protein A antibody. Bacterial cell surface and cytoplasmic protein fractions were examined after growth of Lm-spa^+ ^in BHI containing 1% amberlite XAD-4. Addition of XAD-4 to the culture medium enhances the activity of the virulence gene activator PrfA and hence leads to an enhanced transcription of the *spa *gene which is under the control of the PrfA-dependent *hly *promoter [25]. SPA was readily detected in the cell surface protein fraction of Lm-spa^+ ^and to a lower extent in the internal protein extract fraction. (Figure 1A). As expected, no SPA was present in the parental strain Δ*trpS,aroA,inlA/B *× pFlo-*trpS*, termed Lm-spa^- ^(Figure 1A).

### Fc-mediated binding of antibodies to SPA on the surface of *L. monocytogenes*

The functionality of Fc-mediated binding of antibodies to SPA on the surface of Lm-spa^+ ^was first tested by immunofluorescence microscopy of Lm-spa^- ^and Lm-spa^+ ^after incubation of the bacteria with polyclonal rabbit antibodies directed against ovalbumin (OVA). After addition of FITC-conjugated OVA no fluorescence was detected with Lm-spa^-^, while the Lm-spa^+ ^strain showed a strong fluorescence (Figure 1B).

A more quantitative analysis of SPA expression was performed by flow cytometry after staining Lm-spa^+ ^and Lm-spa^- ^with FITC-conjugated rabbit-anti-goat-antibodies. Lm-spa^- ^bacteria showed no staining while the Lm-spa^+ ^bacteria were stained almost completely (Figure 1C).

In addition, the number of SPA molecules per bacterial cell was determined indirectly. For this goal Lm-spa^+ ^was incubated simultaneously with a primary antibody against native albumin as model protein in the presence of an excess of albumin. The bacteria bound the albumin-loaded antibody to their surface via SPA and later on the amount of bound protein was quantified. Therefore the samples were denatured in Laemmli buffer and size separated by SDS-PAGE. Subsequently the amount of bound albumin was determined by Western blot analysis using a primary antibody recognizing denatured albumin. Untreated Lm-spa^+ ^did not bind albumin, while Lm-spa^+ ^coated with the albumin-specific antibody bound albumin (Figure 1D).

Computer aided comparison of the band intensity of bacterially bound albumin with the known protein amount of the positive control revealed a 7 times higher signal intensity. Thus 70 ng albumin were bound to 5 × 10^8 ^bacterial cells. With albumin having a protein mass of 69 kDa 70 ng correspond to 8,73*10^9 ^molecules. Divided by the number of bacteria employed for the coating (5*10^8 ^CFU) approximately 120 albumin molecules were bound per bacterial cell. Assuming two bound albumin molecules per antibody and one antibody per SPA molecule, this means that at least 60 SPA molecules are exposed in the correct orientation on the surface of each Lm-spa+ cell.

### Internalization of antibody coated Lm-spa^+ ^into cancer cell lines expressing the respective antibody ligand

After the successful demonstration of SPA binding to the bacterial surface, it was important to investigate whether the binding of tumor receptor-specific antibodies to SPA on the surface of Lm-spa^+ ^can mediate specific cell recognition and internalization of the bacteria into the tumor cells. The mouse mammary gland cell line 4T1 (HER1- and HER2 negative) and the isogenic cell line 4T1-HER2 (stably transfected with human-HER2 [26]) were used in these experiments as well as the monoclonal antibodies Cetuximab and Trastuzumab directed against HER1 and HER2, respectively. Both mAbs belong to the same IgG1 subclass of immunoglobulins, but Cetuximab is a mouse/human chimeric antibody whereas Trastuzumab is almost completely humanized. Cetuximab is therefore a control for unspecific antibody coating of Lm-spa^+ ^when analyzing the interaction of these bacteria with murine 4T1-HER2 cells.

**Figure 2:**
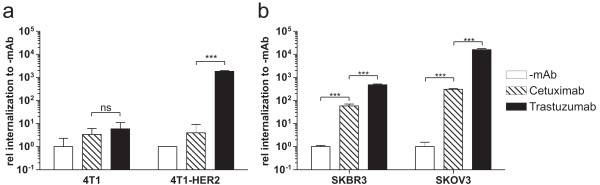
**Internalization of Cetuximab- or Trastuzumab- coated Lm-spa^+ ^relative to uncoated Lm-spa^+ ^(-mAb) into different cell lines**. (**a**) Mouse mammary cancer cell line 4T1, the HER2 transduced isogenic 4T1-HER2 and (**b**) the human mammary/ovary cancer cell lines SK-BR-3 and SK-OV-3, respectively, were infected with Lm-spa^+ ^after coating with different antibodies. Intracellular colony forming units (CFU) were determined after gentamicin treatment by serial plating and the internalization rate of the antibody-coated relative to the uncoated bacteria was calculated.

The *Lm *EGDe wild-type strain was able to efficiently enter both cell lines 4T1 and 4T1-HER2 (data not shown). As expected, the Lm-spa^- ^strain (which is InlAB-negative) was not internalized by 4T1 or 4T1-HER2 cells regardless of whether these bacteria were incubated with Cetuximab or Trastuzumab (Additional file 1a, c). Lm-spa^+ ^was also unable to enter 4T1 and 4T1-HER2 cells without antibody coating or with Cetuximab coating. However, high internalization of Lm-spa^+ ^into 4T1-HER2 cells was observed when these bacteria were coated with Trastuzumab (Figure 2A, Additional file 1e).

Lm-spa^- ^was also not internalized by the human SK-BR-3 and SK-OV-3 cancer cells (both expressing HER1 and HER2) in the presence or absence of the two mAbs (Additional file1b, d). In contrast Lm-spa^+ ^coated with either Cetuximab or Trastuzumab, but not the uncoated Lm-spa^+^, was able to enter these cells efficiently (Figure 2B, Additional file 1f).

As shown in Figure 2A and 2B the coating of Lm-spa^+ ^with the receptor-specific antibody led to a highly significant increase of Lm-spa^+ ^internalization (ranging from 2 × 10^2^- to 10^4^-fold) into tumor cells expressing the respective receptor on the surface. Antibody mediated internalization was followed by bacterial escape into the host cell cytosol and replication as examined by immunofluorescence (Additional file 2).

### Herceptin-mediated internalization of Protein A coated beads into the 4T1-HER2 cell line

**Figure 3:**
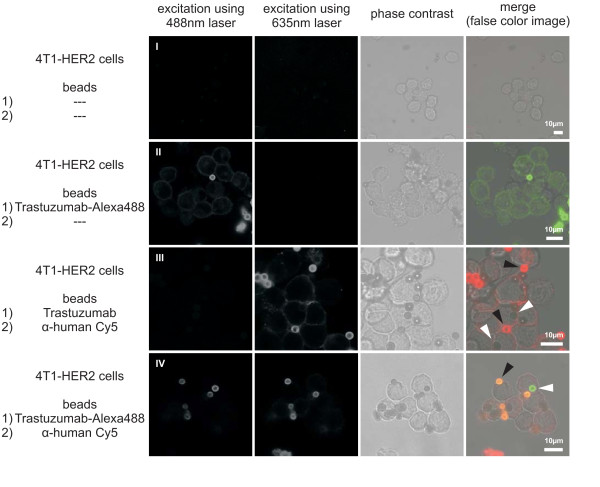
**Internalization of antibody coated Dynabeads Protein A into 4T1-HER2 cells**. The beads were coated with the first antibody (1) and incubated with 4T1-HER2 cells. Following washing, the cells were incubated with the second antibody (2) and analyzed by confocal immunofluorescence microscopy. Beads labeled with (1) are located intracellular, while beads labeled with (1) and (2) are located extracellular. Non coated beads showed no background fluorescence (I) and were efficiently coated with Trastuzumab-Alexa488. On bead-coating with Trastuzumab or Trastuzumab-Alexa488 (II, III) some beads were located in the cell (marked with white arrowheads). Some beads remained outside the cells (marked with black arrowheads). Presence of bead fluorescence was analyzed in image stacks of at least 5 μm thickness to exclude false negatives (Additional file 4).

Trastuzumab coated beads of 2.8 μm diameter were used to assess whether this antibody alone is able to induce internalization of large particles into a cell line expressing the HER2 receptor. Alexa Fluor 488 labeled Trastuzumab (Trastuzumab-Alexa488) was efficiently bound by Dynabeads Protein A (Invitrogen, beads), while the goat α-human Cy5 antibody could not be bound directly (Figure 3, II; Additional file 3). If the beads were preincubated with Trastuzumab or Cetuximab, α-human Cy5 antibody efficiently bound to this antibody, indirectly labeling this beads (Figure 3, III; Additional file 3). Beads depicted in green were labeled with Trastuzumab-Alexa488, while red ones bound α-human Cy5 antibody.

Beads were coated with Trastuzumab-Alexa488 and incubated with 4T1-HER2 cells. Following this incubation Cy5 labeled α-human antibody was added into the supernatant, resulting in a double staining of extracellular beads. Beads without antibody treatment prior to incubation with eukaryotic cells were found to remain completely extracellular (Additional file 4). In contrast some of the beads treated with Trastuzumab or Trastuzumab-Alexa488 were located intracellular (Figure 3, III, IV). Only pretreatment with Trastuzumab and its labeled derivate allowed internalization of beads into this cell line, Cetuximab did not trigger internalization (data not shown). Thus, Trastuzumab is sufficient to mediate internalization of beads, larger than bacteria, into the 4T1-HER2 cell line.

### Serum strongly reduces the internalization of antibody-coated Lm-spa^+^

**Figure 4:**
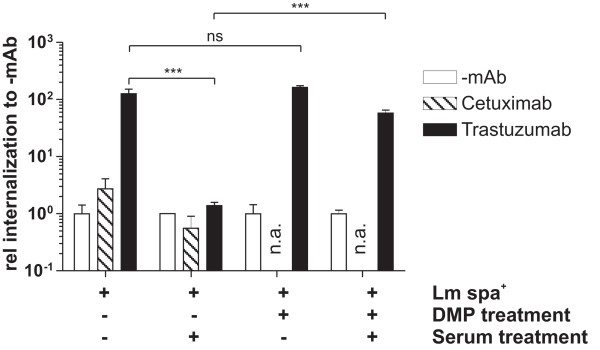
**Effect of serum incubation on antibody-mediated internalization of Lm-spa^+^**. The bacteria were incubated with PBS (-mAb), Cetuximab or Trastuzumab and the antibodies were covalently bound to protein A by crosslinking with DMP. Subsequently the bacteria were incubated with murine serum prior to infection of 4T1-HER2 cells. Intracellular CFU was determined after gentamicin treatment by plating serial dilutions. The relative internalization rate in comparison to uncoated bacteria was calculated and is shown.

For the evaluation of antibody-mediated targeting *in vivo *Lm-spa^+ ^was coated with Trastuzumab and 1 × 10^8 ^bacteria were injected i.v. into Balb/c SCID mice bearing 4T1-HER2 tumors. In a control group equal numbers of uncoated Lm-spa^+ ^were used. In contrast to the *in vitro *data where Lm-spa^+ ^coated with Trastuzumab showed highly significant internalization into 4T1-HER2 cells compared to uncoated Lm-spa^+ ^(Figure 2A), no significant difference of the bacterial counts in liver, spleen or tumor was observed when the mice were treated with antibody-coated or -uncoated Lm-spa^+ ^(Additional file 5). To rule out the possibility that during the blood passage the non-covalently bound mAbs on the surface of the coated Lm-spa^+ ^bacteria might be displaced by the IgG antibodies of the blood serum fresh murine serum was added to Trastuzumab-coated Lm-spa^+ ^bacteria prior to *in vitro *infection of 4T1-HER2 cells. This treatment completely abolished the specific internalization and the coated Lm-spa^+ ^behaved like uncoated Lm-spa^+ ^bacteria (Figure 4).

To prevent the displacement of the SPA-bound antibody by serum antibodies we covalently linked Trastuzumab to SPA on the bacterial surface with Dimethyl pimelinediimidate dihydrochloride (DMP), a homobifunctional imidoester cross-linker. The concentration of DMP and the incubation conditions were evaluated to achieve optimal crosslinking and bacterial viability (data not shown). Treatment of Lm-spa^+ ^with DMP under these conditions did not alter the internalization efficiency significantly, but largely prevented the negative effect of murine serum on the internalization of Trastuzumab-coated Lm-spa^+ ^into 4T1-HER2 cells *in vitro *(Figure 4).

### Targeting of Lm-spa^+ ^coated with covalently bound antibody to 4T1-HER2 tumors in mice

**Figure 5:**
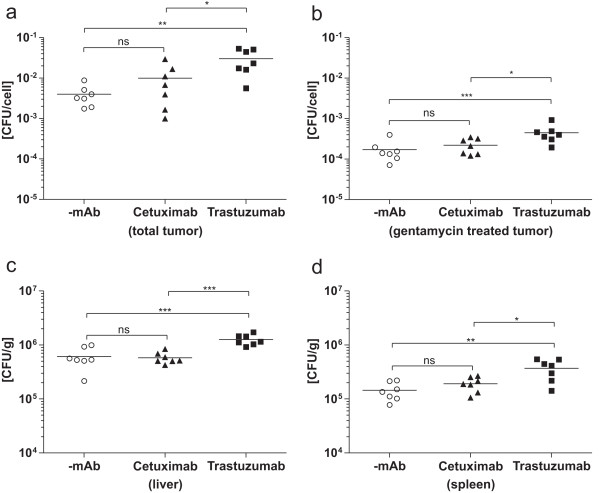
**Antibody-mediated targeting of uncoated (-mAb), Cetuximab- or Trastuzumab- coated Lm-spa^+ ^after antibody crosslinking in xenografted mouse tumor models**. In seven Balb/c SCID mice per group 4T1-HER2 tumors were induced and 14 days later the mice were infected with 1 × 10^8 ^CFU of differently coated Lm-spa^+^. 24 h later mice were sacrificed and tumors, liver and spleen excised aseptically. Tumors were digested with DNAse and Dispase to obtain single cell suspensions which were plated in serial dilutions without (**a**) and with gentamicin treatment (**b**) to determine total and intracellular bacterial counts, respectively. Depicted is the bacterial count per cell in the cell suspension. Liver (**c**) and spleen (**d**) were homogenized and plated in serial dilutions.

The above described *in vitro *data showing that the antibody can be covalently linked to SPA on the surface of Lm-spa^+ ^without losing the bacterial viability encouraged us to modified antibody-targeted bacteria in the mouse tumor model system. Briefly, Balb/c SCID mice carrying 4T1-HER2 tumors were injected i.v. with 1 × 10^8 ^Lm-spa^+ ^bacteria coated with Trastuzumab crosslinked to SPA. Similarly treated Cetuximab-coated Lm-spa^+ ^bacteria were included in this *in vivo *experiment as a negative control. One day after infection the bacterial counts were determined in liver, spleen and tumor. For the distinction of intra- and extracellularly replicating bacteria, the tumor tissue was enzymatically digested to obtain a single cell-suspension, part of which was treated with gentamicin to kill the extracellular bacteria while the other part remained untreated to allow the determination of the total bacterial counts in the tumor. Both fractions were plated in serial dilutions to obtain viable bacterial counts (CFU). As shown in Figure 5 injection of tumor bearing mice with Lm-spa^+ ^coated with covalently bound Trastuzumab resulted in significantly increased CFU per cell of tumor tissue compared to Lm-spa^+ ^with covalently bound Cetuximab and uncoated Lm-spa^+ ^(Figure 5). This difference was observed in the gentamicin treated as well as in the untreated fractions but the increase is more pronounced in the untreated fractions. The coating with Trastuzumab increased the amount of bacteria 8- to 10-fold, while the amount of intracellular bacteria was elevated only 3- to 4-fold (Figure 5). In liver and spleen a 2-fold increase of bacteria was observed with the Trastuzumab-coated but not with the Cetuximab-coated Lm-spa^+^.

## Discussion

In this study we describe a novel approach for cell targeting which uses an InlA- and InlB- deficient *Lm *mutant expressing SPA anchored to the cell wall. Antibodies bind to these bacteria via their Fc part thereby enabling interaction of the bacteria with receptors (or other ligands) exposed on the surface of target cells recognized by the antibodies. In spite of a relatively low coverage of the bacterial surface with SPA-bound antibodies, a highly efficient targeting of the bacteria to the antibody-recognized tumor cell receptors (ligands) is observed. Two clinically approved humanized and chimeric monoclonal antibodies, Trastuzumab and Cetuximab, respectively, directed against the cell surface receptors HER2/neu and EGFR/HER1 respectively, were applied in this study. These receptors are overexpressed in several types of cancer, thus representing excellent specific targets for *Lm *coated with Trastuzumab or Cetuximab [27,28]. Antibody coated *Lm *strains not only showed specific binding to tumor cell lines but also a highly efficient internalization into tumor cell lines. This internalization was clearly independent of the known InlA and/or InlB-mediated invasion machinery of *Lm*, as these two major invasion factors [reviewed in 18] were deleted in the antibody-coated *Lm *strains. Experiments showing internalization of Trastuzumab-coated beads into HER2 expressing cells indicate that the internalization may be completely independent of listerial virulence factors. The bacteria may be taken up by the host cell passively, as a consequence of receptor recycling. The cellular recycling rate of the EGF-family receptors has been shown to increase upon ligand interaction and antibody-mediated dimerization [29]. After Trastuzumab- mediated internalization *Lm *was able to escape into the cytosol, replicate and spread to adjacent cells as demonstrated by immunofluorescence. The efficiency of these intracellular steps was comparable to that of the corresponding Δ*aroA *attenuated wild-type strain.

Transfer of antibody-mediated targeting into xenograft mouse tumors was initially unsuccessful. Subsequent *in vitro *experiments revealed that the incubation of the antibody coated bacteria with murine serum completely abrogated the specific internalization, but this effect was largely prevented by crosslinking of the antibody to SPA on the surface of live bacteria.

Crosslinking enabled also the targeting of the antibody-coated bacteria to a 4T1-HER2 xenograft mouse tumor. The number of Trastuzumab-coated bacteria in the tumor tissue increased 8 to 10-fold when compared to uncoated bacteria. Although less than 5% of these bacteria were intracellular, the bacterial count was significantly increased relative to bacteria not coated with Trastuzumab. This 3-fold increase in the number of intracellular bacteria was antibody specific, since bacteria coated with a second antibody (Cetuximab), that recognizes the related receptor EGFR, did not show a significant increase compared to uncoated bacteria.

The bacterial counts in liver and spleen were 2-fold increased with the Trastuzumab-coated *Lm *compared to the uncoated bacteria, while the Cetuximab-coated bacteria colonized liver and spleen with a similar efficiency as the uncoated ones. The humanized Trastuzumab contains a larger portion of non-mouse peptide sequences than the human/mouse chimeric Cetuximab. Thus a stronger immune reaction against Trastuzumab might lead to an enhanced uptake of bacteria coated with Trastuzumab by phagocytic cells in liver and spleen.

Recently Bereta and coworkers [23] described an alternative approach of antibody-mediated targeting of bacteria whereby a single chain antibody (scFv) was expressed by *Salmonella *VNP20009. In this study the scFv directed against CEA caused only a low increase (about 2-fold) of the targeted bacteria in a CEA overexpressing tumor seven days post infection. Our approach, which crosslinks the antibody to the surface-exposed SPA, shows not only a better uptake of the targeted bacteria by the tumor (already 24 h post intravenous injection), but is also more versatile, since it requires only a specific antibody against a cell surface-exposed ligand to specifically target the bacteria to the ligand-producing cells. Whether these bacteria will be subsequently internalized by the target cells will presumably depend on the cell receptor recognized by the antibody.

## Conclusions

Certainly, further studies are needed to test this promising cell targeting technology for possible therapeutic applications (e.g. drug delivery to selected cells) but the experiments shown here successfully demonstrate the proof of principle of the approach.

## Methods

### Ethics Statement

All animals experiments were carried out in accordance with protocols approved by the Regierung von Unterfranken, Germany.

### Bacterial strains, plasmids, media and growth conditions

All strains and plasmids used are listed in Table 1. *E.coli *DH10b was used for all plasmid DNA manipulations. Competent *Lm *cells were prepared and transformed by electroporation as described by Park and Stewart [30]. All experiments were performed with *Lm *grown to mid-logarithmic growth phase (OD_600 _= 0.8) at 37°C cultivated in brain heart infusion (BHI, BD Difco, USA). In experiments indicated, addition of amberlite XAD-4 to the BHI media led to the upregulation of SPA expression in mid-logarithmic phase by activating PrfA and thus listeriolysin promoter P*_hly_*. Bacteria were washed twice in 0.9% NaCl (Applichem, Germany) solution, resuspended in 20% v/v glycerol (Applichem, Germany) in 0.9% NaCl solution and stored as aliquots at -80°C. Bacterial CFUs were determined by plating serial dilutions on BHI agar plates supplemented with 5 μg/ml tetracycline (Sigma, Germany).

**Table 1:** Bacterial strains and plasmids

Strains and plasmids	Relevant genotype	Reference or source
***L. monocytogenes***		
EGD-e Δ*trpS *× pFlo-*trpS*	*wild-type*	T. Chakraborty (University of Giessen, Germany [36]
ΔtrpS,inlA/B × pFlo-trpS		[32]
Lm-spa^-^	ΔtrpS,aroA,inlA/B × pFlo-trpS	This work
Lm-spa^+^	ΔtrpS,aroA,inlA/B,int::P_hly_-spa × pFlo-trpS	This work
ΔtrpS × pSP0-P_actA_-gfp		[36]
Lm-spa^- ^× pSP0-P*_actA_-gfp*	ΔtrpS,aroA,inlA/B × pSP0-P_actA_-gfp	This work
Lm-spa^+^,aroA^+ ^× pSP0-P*_actA_-gfp*	ΔtrpS,inlA/B,int::P_hly_-spa × pSP0-P_actA_-gfp	This work
Lm-spa^+ ^× pSP0-P*_actA_-gfp*	ΔtrpS,aroA,inlA/B,int::P_hly_-spa × pSP0-P_actA_-gfp	This work

**Plasmids**		
pFlo-*trpS*	Tc^R^,	[36]
pSP0-P_actA_-gfp	Em^R^, *gfp*-ORF, *actA-*promoter	[36]
pLSV101intAB	Em^R^, ORI^ts^, mutagenesis plasmid	[31]
pLSV101intAB::P*_hly_*-*spa*	*spa*-ORF, *hly-*promoter	This work

### Plasmid and strain construction

To amplify the *spa *gene from *S. aureus *strain 8325 wt, genomic DNA was used in a PCR reaction using the specific primers proteinA forward (5'-GCAAATGCATCGCAACACGATG-3') and proteinA reverse (5'-TTTTCCCGGGCGTTGTGTATTGTTT-3'). The DNA fragment was cut with SmaI and cloned into the vector pUC18, leading to the plasmid pUC18-*spa*. The fragment was then cut and cloned into the plasmid pUC18-P*hly *using the NsiI and XmaI restriction sites. The fragment P*hly-spa *was PCR amplified by the Primers M13 universe 2 (5'-GTAAAACGACGGCCATGGC-3') and M13 rev (5'-CAGGAAACAGCTATGAC-3') to introduce a NcoI restriction site. The fragment was then cloned into plasmid pLSV101-intAB [31] using the restriction sites NcoI and SacI. The resulting plasmid pLSV101-intAB::P*hly-spa *was transformed into *L. monocytogenes *Δ*trpS,inlA/B *× pFlo-*trpS *[32] and *L. monocytogenes *Δ*trpS,aroA,inlA/B *× pFlo-*trpS *[aroA attenuated as described in 33] and a homologous recombination technique was used to construct a deletion mutant [34]. Because *trpS bearing *plasmids are fully stable in the Δ*trpS *mutant without the addition of antibiotics this strain was used for mutant generation.

### Western blot analysis

*L. monocytogenes *protein extracts were prepared as described [35]. Surface proteins were extracted by incubation in 1% sodium dodecyl sulfate (SDS) for 20 min. Blotted proteins were probed with a polyclonal goat antibody against Protein A (Biomeda, CA, USA) or polyclonal rabbit antibody against murine serum albumin (ab19196 - abcam, UK). Secondary Peroxidase-conjugated antibodies and ECL Western blot detection reagent (Amersham Biosciences, Germany) were used for visualization of bands.

### Analysis of bacterial protein A surface expression

Bacteria were washed in PBS and incubated for 1 h at 25°C with polyclonal FITC-conjugated rabbit-anti-goat immunoglobulin G (H+L, Sigma, Germany) for flow cytometry or polyclonal rabbit antibody directed against ovalbumin (C6534, Sigma, Germany) for immunofluorescence microscopy. Controls were incubated with PBS. Bacteria were washed 2-3 times with PBS and analyzed using an Epics XL flow cytometer (Beckman Coulter) or further incubated with FITC-conjugated OVA (Molecular Probes, Germany). After repeated washing, bacteria were loaded on microscope slides and analyzed by fluorescence microscopy (Leica, Germany).

### Antibody-coating, crosslinking and serum treatment of *L. monocytogenes*

For antibody-coating, 5 × 10^8 ^CFU were washed with PBS (pH 8.2) and resuspended in 100 μl PBS containing 2.5 μg of Cetuximab (Merck, Germany) or 2.37 μg of Trastuzumab (Roche, Germany), respectively. Alexa Fluor labeled antibodies were generated using the Apex Antibody labeling kit (Invitrogen) following the manufacturers guidelines. The bacteria were incubated under vigorous shaking for 45 min at room temperature (RT). Bacteria were washed with PBS (pH 8.2) and diluted for further use.

Crosslinking of antibodies to SPA on the surface of Lm-spa^+ ^was performed using dimethyl pimelinediimidate dihydrochloride (DMP, Biochemika Fluka, Germany). Freshly prepared DMP in PBS (pH 8.2) was added at a final concentration of 0.65 mg/ml to the antibody coating reaction. After 45 min the bacteria were washed with PBS (pH 8.2) and the crosslinking procedure was repeated for additional 45 min with the same concentration of DMP. Control bacteria were treated likewise without antibody addition.

Serum treatment of bacteria was performed after coating and crosslinking prior to infection. Bacteria were mixed with fresh serum from naïve mice and incubated for 1 h under vigorous shaking at RT, washed with PBS (pH 8.2) and finally diluted.

The amount of SPA per bacterial cell was determined by Western blot analysis. 5 × 10^8 ^CFU were resuspended in 100 μl PBS and 0.1 μg of anti mouse albumin antibody (Abcam ab34807, UK) and 200 ng of serum albumin (Sigma, Germany) were added. The suspension was incubated under vigorous shaking for 45 min at RT. Bacteria were washed three times with 0.05% Tween 20 in PBS and analyzed by SDS-PAGE and Western blotting.

### Handling of Dynabeads Protein A

Dynabeads Protein A (Invitrogen, Germany) were coated with Trastuzumab following the manufacturer's protocol. 1.2 × 10^5 ^4T1-HER2 cells were seeded on cover slips in 24-well plates and incubated with antibody-labeled and non-labeled beads. 25 μg beads were added per well in culture medium lacking FCS. Cells were incubated 1 h at 37°C and with 5% CO_2_. The coverslips were washed in PBS and fixed in 4% PFA for 10 minutes at room temperature. After washing using PBS, the cells were incubated with the second antibody (α-human Cy5, Abcam ab6561, UK) for 1 h at room temperature in the dark. Following an additional washing step in PBS the cover slips were embedded and analyzed by immunofluorescence microscopy.

### Cell culture and infection experiments

4T1 cells (mouse mammary gland tumor cell line; ATCC/Promochem, Germany) were cultured in RPMI 1640 medium. 4T1-HER2 cells (mouse mammary gland tumor cell line transduced with human HER2, [26]) were cultured in DMEM medium. SK-BR-3 (human mammary adenocarcinoma cell line, ATCC Promochem, Germany) and SK-OV-3 (human ovary adenocarcinoma; ATCC Promochem, Germany) cells were cultured in McCoy's medium. All media (GIBCO) were supplemented with 10% FCS (PAN, Germany) and cultures were kept under a 5% CO_2 _atmosphere at 37°C. If not stated otherwise, infection of cell lines was performed with 100 bacteria per cell (MOI 100) as described earlier [14]. Briefly 1.2*10^4 ^cells were seeded at least 16 h before infection and washed in medium lacking FCS directly before infection. The infection was performed in medium lacking FCS for 1 h and followed by 1 h incubation with medium containing 10% FCS and 100 μg/ml gentamicin to kill extracellular bacteria. Cells were then lysed in 0.1% Triton-X100 and plated in serial dilutions on agar plates containing the appropriate antibiotics for selection.

### Animal handling and *in vivo *experiments

Six to eight weeks old, female Balb/c SCID mice were purchased from Harlan, Germany. Xenograft tumor growth was induced by injecting 5 × 10^4 ^4T1-HER2 cells into each flank of shaven abdominal skin. When tumors reached 6 mm in diameter, animals were randomized and divided into three equal groups. 1 × 10^8 ^bacteria were injected into the lateral tail vein and 24 h post infection mice were sacrificed. Liver, spleen and tumors were excised and the organ weight was determined. Liver and spleen were homogenized in 1 ml PBS and serial dilutions were plated for CFU determination. Tumors were digested for 30-45 min at 37°C and 5% CO_2 _under 100 u/ml DNAse (Sigma, Germany) and 2 μg/ml Dispase (Gibco Invitrogen, Germany) treatment and homogenized with 70 μm and 40 μm cell strainers. Cell counts were determined in a Fuchs-Rosenthal counting chamber. One part of the cells was treated for 1 h at 37°C with 100 μg/ml gentamicin to kill extracellular bacteria, while the other part was left untreated. Cells were washed twice in PBS and finally lysed in 0.1%Triton-X100 for CFU determination by plating serial dilutions. The CFU in the tumors was normalized to the number of cells in the homogenized tumor tissue. The CFU of liver and spleen was normalized to the organ weight.

### Experimental design and statistical analysis

All experiments were conducted at least three times with duplicate samples; a representative experiment is shown. In invasion experiments the CFU was arbitrarily set on the detection limit if no colonies were visible on the agar plates. Statistical evaluation was performed on logarithmized data by two-sided students T-test; p-values larger than 0.05 were labeled with 'ns', p-values of p < 0.05 were marked with '*', p-values of p < 0.005 were marked with '**' and p-values of p < 0.001 were marked with '***'. Differences marked with asteriks were considered as significant.

## Competing interests

The authors declare that they have no competing interests.

## Authors' contributions

MH and AF performed the study; MH, AF, BB, KG, IG, CH, CS, JS, JF, URR and WG performed the analysis and MH, AF, URR and WG wrote the manuscript. All authors approved the final manuscript.

## Supplementary Material

Additional file 1 **Internalization of Cetuximab- or Trastuzumab- coated Lm-spa^- ^relative to uncoated Lm-spa^- ^(-mAb) into different cell lines**. (**a**) Mouse mammary cancer cell line 4T1, the HER2 transduced isogenic 4T1-HER2 and (**b**) the human mammary/ovary cancer cell lines SK-BR-3 and SK-OV-3, respectively, were infected with a MOI of 100 with Lm-spa^- ^after coating with different antibodies. Intracellular colony forming units (CFU) were determined after gentamicin treatment by serial plating and the internalization rate of the antibody-coated relative to the uncoated bacteria was calculated. As expected, the Lm-spa^- ^strain (which is InlAB-negative) was not internalized by 4T1, 4T1-HER2, SKBR3 or SKOV3 cells regardless whether these bacteria were incubated with Cetuximab or Trastuzumab. Raw CFU data of intracellular bacteria used for calculation of (**a**) and (**b**) is shown in (**c**) and (**d**). Raw CFU data of intracellular bacteria used for calculation of Figure 2a and Figure 2b is shown in (**e**) and (**f**). Click here for file

Additional file 2 **Immunofluorescence microscopy showing the replication of *Lm-spa^+ ^*in the cytosol of SK-BR-3 cells**. SK-BR-3 cells were infected at a MOI 10 with *L. monocytogenes *strains Δ*trpS *× pSP0-P*_actA_-gfp *(**a**), *Lm-spa^- ^*× pSP0-P*_actA_-gfp *(**b**) and *Lm-spa*^+ ^× pSP0-P*_actA_-gfp *(**c**) preincubated with 1 × PBS (i-iii) or Trastuzumab (iv-vi) and GFP-expression was monitored by fluorescence microscopy at the indicated time points. Bright field and fluorescence overlay images are shown. The *L. monocytogenes *control strain Δ*trpS *× pSP0-P*_actA_-gfp *shows the typical intracellular life cycle independent of preincubation with Trastuzumab (**a**). *L. monocytogenes *strain *Lm-spa^- ^*× pSP0-P*_actA_-gfp *is unable to infect SK-BR-3 cells as expected (**b**). *L. monocytogenes *strain *Lm-spa*^+ ^× pSP0-P*_actA_-gfp *infects cells and replicates in the cytosol only after preincubation with Trastuzumab (**c**). Because of the *aroA *deletion *Lm-spa*^+ ^× pSP0-P*_actA_-gfp *hardly spreads to neighboring cells. Click here for file

Additional file 3 **Examination of antibody binding to Dynabeads Protein A**. Beads were incubated with fluorescently labeled antibodies, washed intensively to remove excess antibodies, and investigated by confocal immunofluorescence microscopy. Beads were incubated simultaneously with the antibodies indicated on the left following bead-manufacturers protocol. Dynabeads Protein A bind efficiently humanized Trastuzumab (II), while no direct binding of goat α-human Cy5 antibody occurs (III). Following pretreatment with the chimeric murine Cetuximab (IV) or Trastuzumab (not shown), the α-human antibody can be bound by the beads (IV, V). Click here for file

Additional file 4 **Absence of Dynabeads Protein A internalization into 4T1-HER2 cells following incubation with goat α-human Cy5 antibody**. Following fixation extracellular beads were counterstained by adding Trastuzumab-Alexa488 into the supernatant. Cells were then analyzed for bead immunofluorescence using a confocal microscope. Stacked images of 5 to 16 μm tissue height were analyzed for Cy5-positive and Alexa488-negative beads. No intracellular beads were detected, indicating the lack of intrinsic bead uptake by 4T1-HER2 cells. Click here for file

Additional file 5 **Antibody-mediated targeting of uncoated (-mAb) or Trastuzumab- coated Lm-spa^+ ^in a xenograft mouse tumor model**. In Balb/c SCID mice 4T1-HER2 cells were injected *s.c*. to initiate tumor growth. 14 days later the mice were infected *i.v*. with 1 × 10^8 ^CFU of differently coated Lm-spa^+^. After 24 h mice were sacrificed and tumors, liver and spleen excised aseptically. Organs were homogenized and plated in serial dilutions. In tumor, liver and spleen no significant differences in the bacterial counts were detected between the uncoated and Trastuzumab coated Lm-spa^+^. Click here for file
