# Supplementary figures and images for: Specific antibody-receptor interactions trigger InlAB-independent uptake of listeria monocytogenes into tumor cell lines
Source: BMC Microbiol. 2011 Jul 11;11:163. doi: 10.1186/1471-2180-11-163 (PMC3142209; doi:10.1186/1471-2180-11-163)

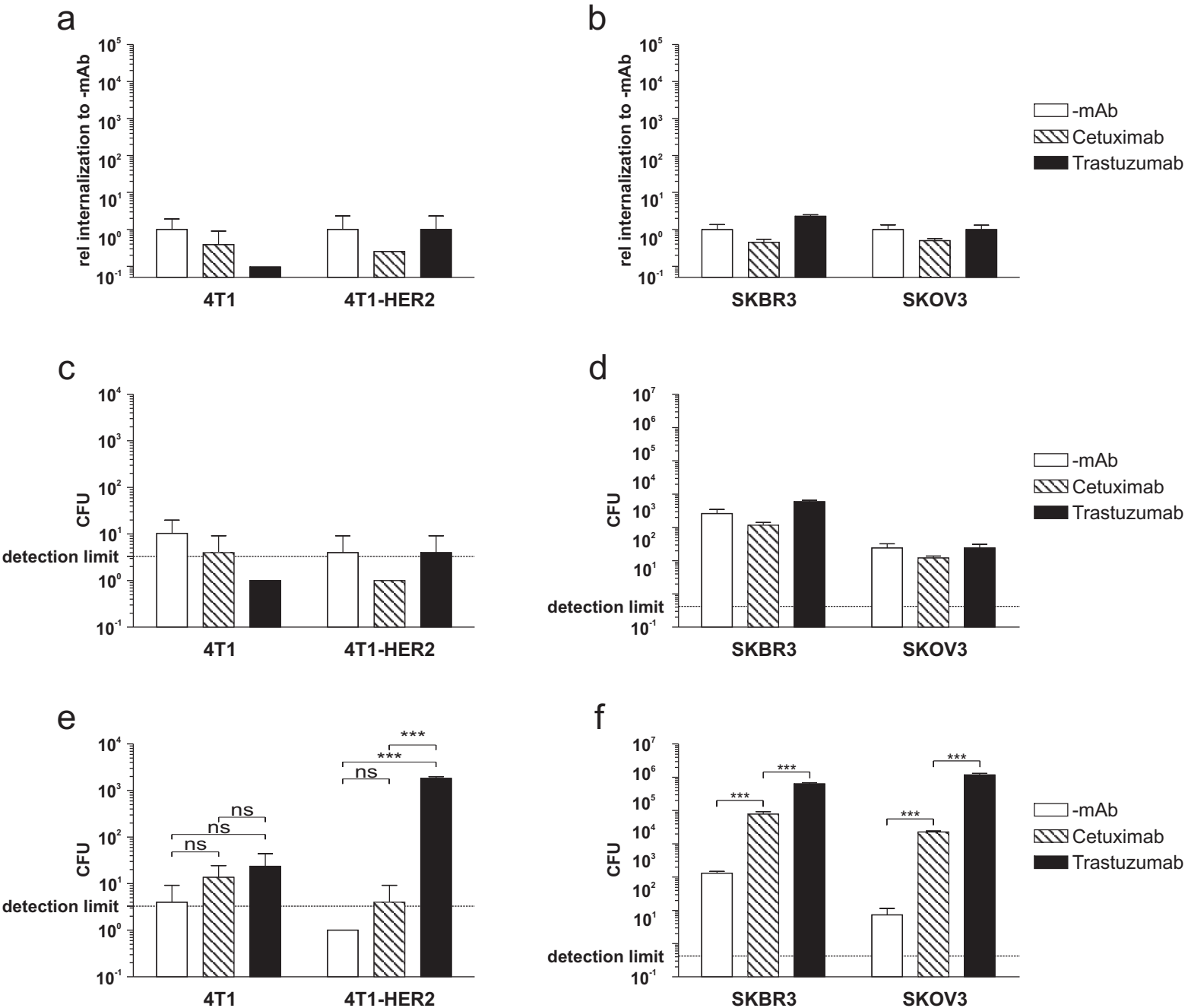

Supplement: Additional file 1 — Internalization of Cetuximab- or Trastuzumab- coated Lm-spa- relative to uncoated Lm-spa- (-mAb) into different cell lines. (a) Mouse mammary cancer cell line 4T1, the HER2 transduced isogenic 4T1-HER2 and (b) the human mammary/ovary cancer cell lines SK-BR-3 and SK-OV-3, respectively, were infected with a MOI of 100 with Lm-spa- after coating with different antibodies. Intracellular colony forming units (CFU) were determined after gentamicin treatment by serial plating and the internalization rate of the antibody-coated relative to the uncoated bacteria was calculated. As expected, the Lm-spa- strain (which is InlAB-negative) was not internalized by 4T1, 4T1-HER2, SKBR3 or SKOV3 cells regardless whether these bacteria were incubated with Cetuximab or Trastuzumab. Raw CFU data of intracellular bacteria used for calculation of (a) and (b) is shown in (c) and (d). Raw CFU data of intracellular bacteria used for calculation of Figure 2a and Figure 2b is shown in (e) and (f). [file 1471-2180-11-163-S1.PDF]

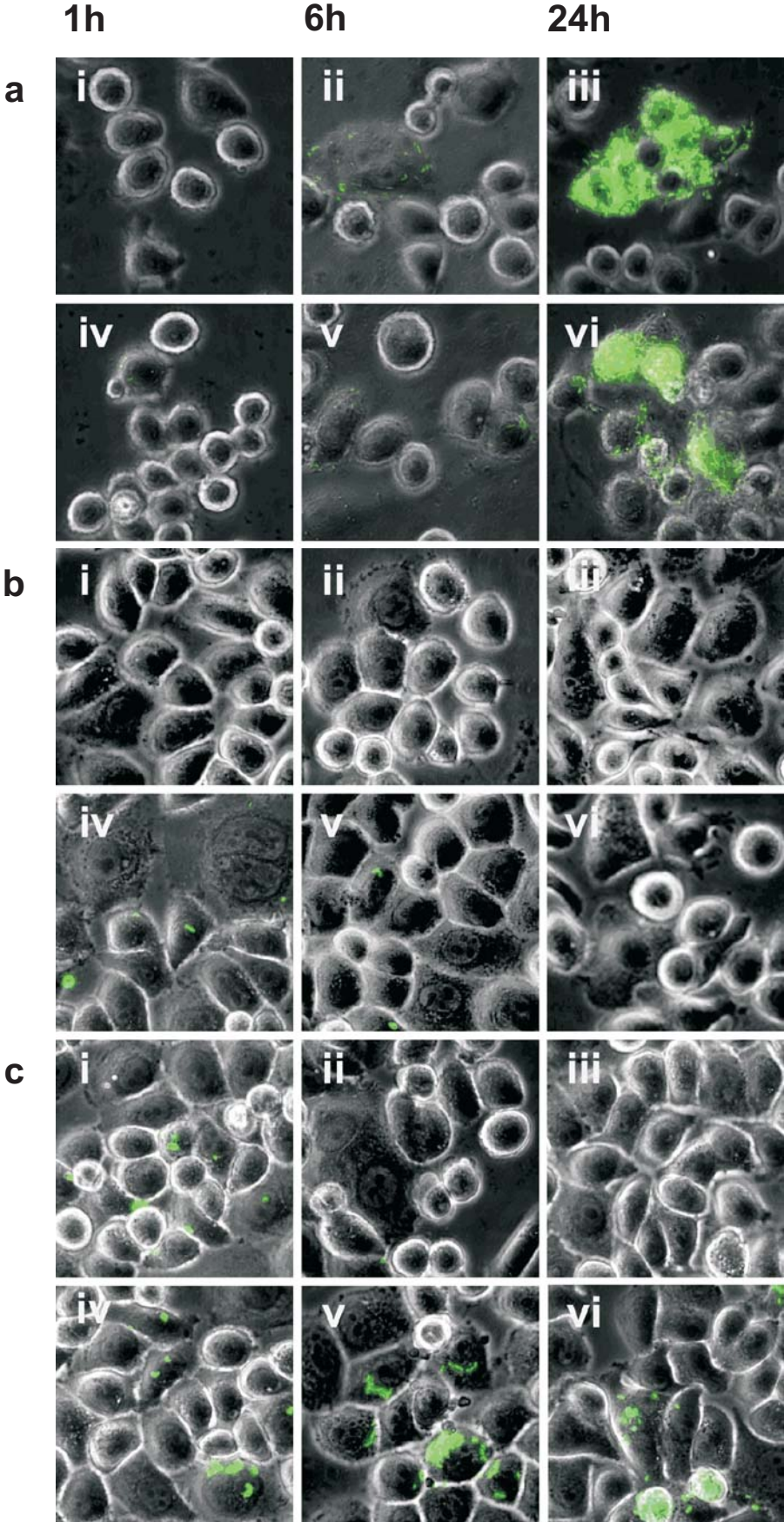

Supplement: Additional file 2 — Immunofluorescence microscopy showing the replication of Lm-spa+ in the cytosol of SK-BR-3 cells. SK-BR-3 cells were infected at a MOI 10 with L. monocytogenes strains ΔtrpS × pSP0-PactA-gfp (a), Lm-spa- × pSP0-PactA-gfp (b) and Lm-spa+ × pSP0-PactA-gfp (c) preincubated with 1 × PBS (i-iii) or Trastuzumab (iv-vi) and GFP-expression was monitored by fluorescence microscopy at the indicated time points. Bright field and fluorescence overlay images are shown. The L. monocytogenes control strain ΔtrpS × pSP0-PactA-gfp shows the typical intracellular life cycle independent of preincubation with Trastuzumab (a). L. monocytogenes strain Lm-spa- × pSP0-PactA-gfp is unable to infect SK-BR-3 cells as expected (b). L. monocytogenes strain Lm-spa+ × pSP0-PactA-gfp infects cells and replicates in the cytosol only after preincubation with Trastuzumab (c). Because of the aroA deletion Lm-spa+ × pSP0-PactA-gfp hardly spreads to neighboring cells. [file 1471-2180-11-163-S2.PDF]

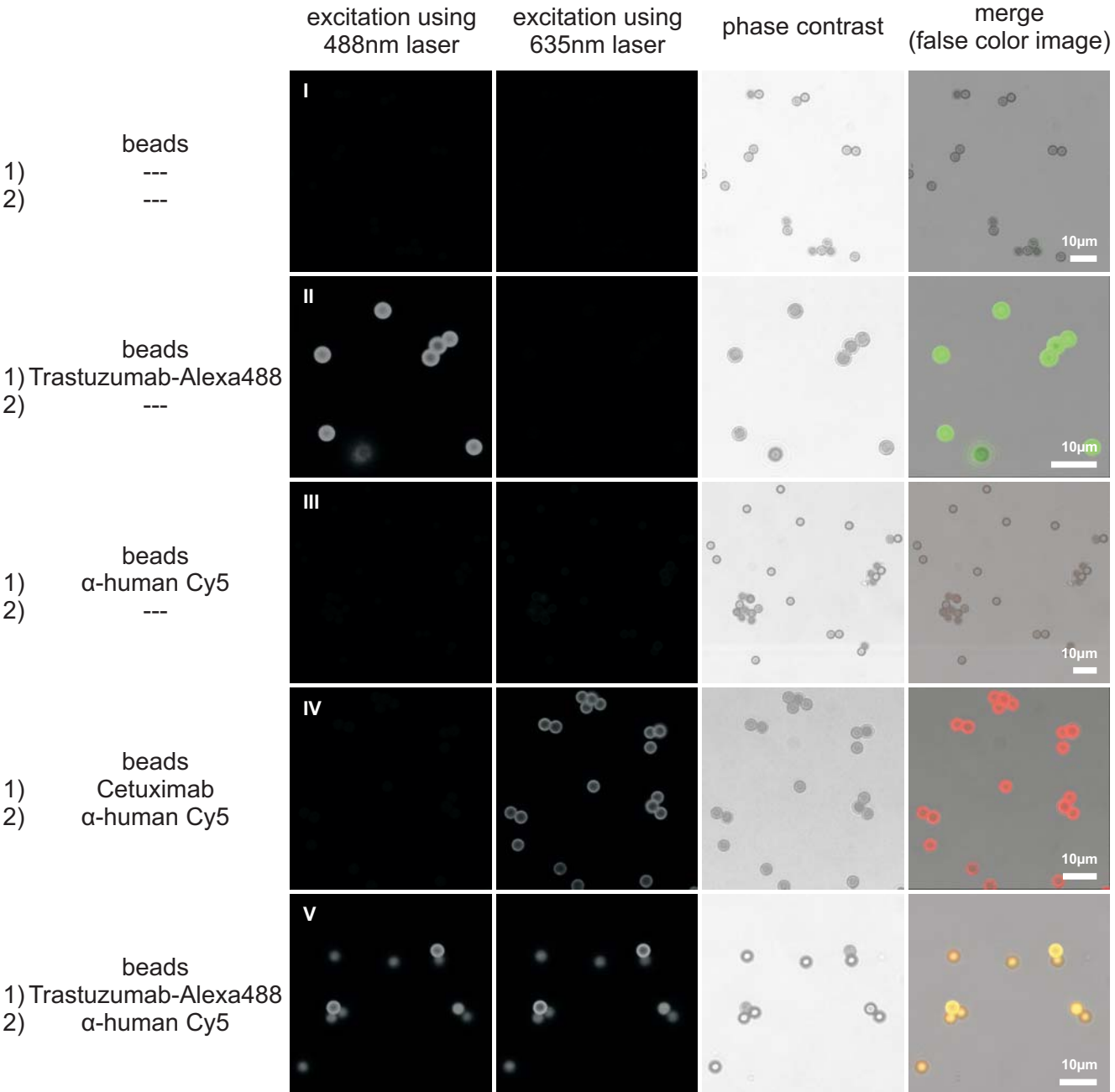

Supplement: Additional file 3 — Examination of antibody binding to Dynabeads Protein A. Beads were incubated with fluorescently labeled antibodies, washed intensively to remove excess antibodies, and investigated by confocal immunofluorescence microscopy. Beads were incubated simultaneously with the antibodies indicated on the left following bead-manufacturers protocol. Dynabeads Protein A bind efficiently humanized Trastuzumab (II), while no direct binding of goat α-human Cy5 antibody occurs (III). Following pretreatment with the chimeric murine Cetuximab (IV) or Trastuzumab (not shown), the α-human antibody can be bound by the beads (IV, V). [file 1471-2180-11-163-S3.PDF]

4T1-HER2 cells  
beads  
1)  $\alpha$ -human Cy5  
2) Trastuzumab Alexa488

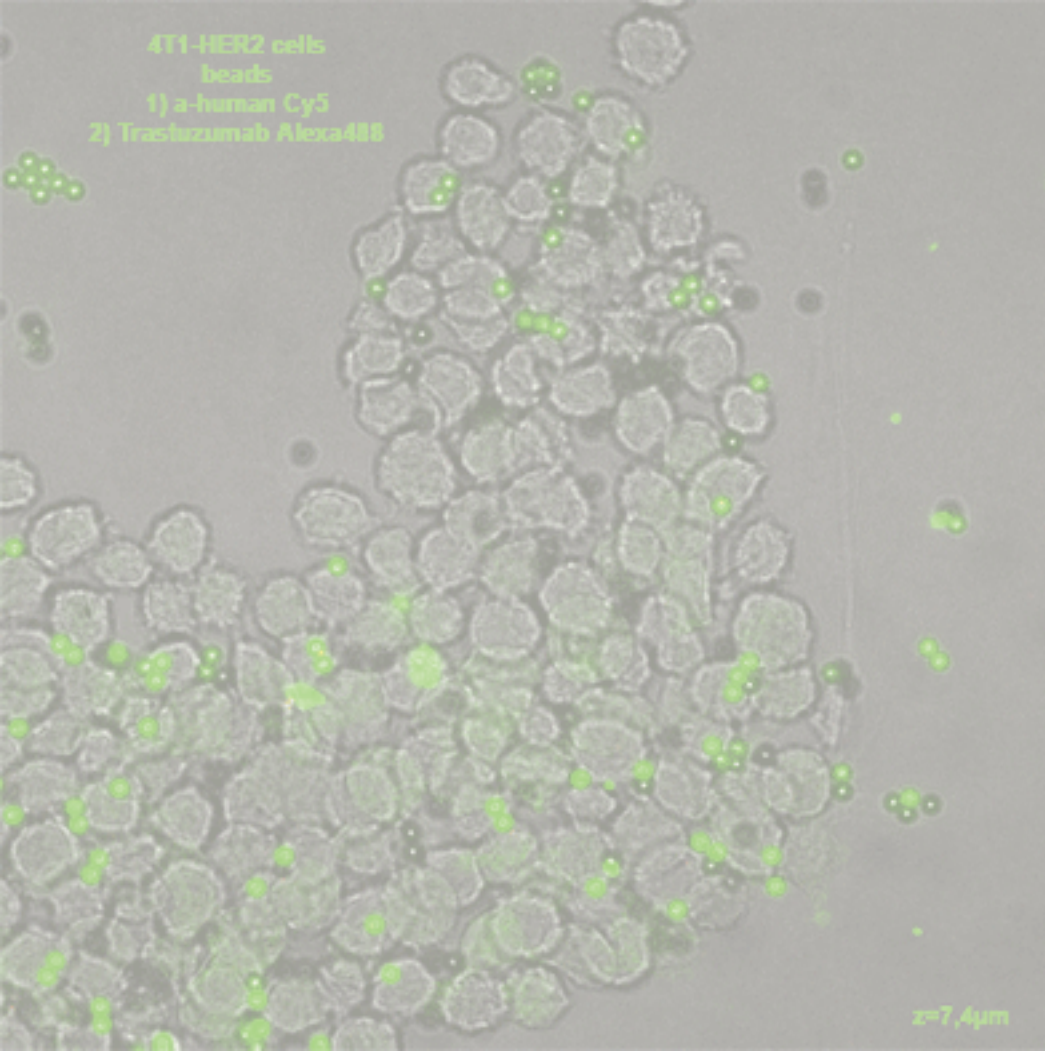

$z=7,4\mu\text{m}$

Supplement: Additional file 4 — Absence of Dynabeads Protein A internalization into 4T1-HER2 cells following incubation with goat α-human Cy5 antibody. Following fixation extracellular beads were counterstained by adding Trastuzumab-Alexa488 into the supernatant. Cells were then analyzed for bead immunofluorescence using a confocal microscope. Stacked images of 5 to 16 μm tissue height were analyzed for Cy5-positive and Alexa488-negative beads. No intracellular beads were detected, indicating the lack of intrinsic bead uptake by 4T1-HER2 cells. [file 1471-2180-11-163-S4.PDF]

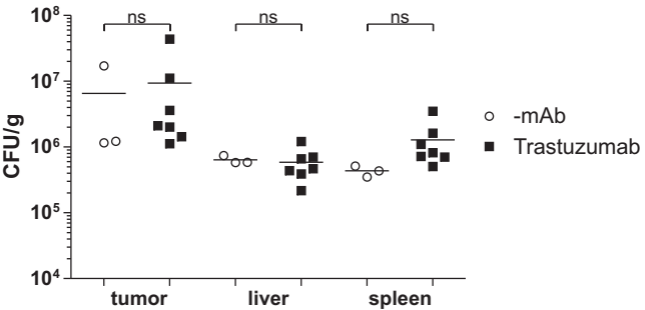

Supplement: Additional file 5 — Antibody-mediated targeting of uncoated (-mAb) or Trastuzumab- coated Lm-spa+ in a xenograft mouse tumor model. In Balb/c SCID mice 4T1-HER2 cells were injected s.c. to initiate tumor growth. 14 days later the mice were infected i.v. with 1 × 10^8^ CFU of differently coated Lm-spa+. After 24 h mice were sacrificed and tumors, liver and spleen excised aseptically. Organs were homogenized and plated in serial dilutions. In tumor, liver and spleen no significant differences in the bacterial counts were detected between the uncoated and Trastuzumab coated Lm-spa+. [file 1471-2180-11-163-S5.PDF]
